# Special Meeting of the Erie County Medical Society, August 4, 1863

**Published:** 1863-08

**Authors:** J. F. Miner

**Affiliations:** Sec’y pro tem.


### SPECIAL MEETING OF THE ERIE COUNTY MEDICAL SOCIETY, AUGUST 4, 1863.

The President, Dr. Charles Winne, communicated the action of the Court upon the suit of Dr. Bartlett for membership in the Society, and read the following reasons assigned by the Court for granting peremptory mandamus:

In Supreme Court, Erie Special Term, March, 1863—

N. Davis, Justice, &c.

The People, ex. rel.

Frederick W. Bartlett, against

The Medical Society of the County of Erie.

Motion for peremptory mandamus. J. A, Allen for Relator; H. W. Rogers for Respondent.

Davis, J.—"I am led to my reflections on this case to the conclusion that the application of the relator was improperly rejected. It is true that he had been guilty of acts of gross empiricism in the publication of the advertisement annexed to his affidavit; and had that publication been continued at the time of his application I should regard the action of the Society as entirely justifiable. But it appears without contradiction that it had been abandoned for years, and that the practice of the relator had been changed from the *specialty* mentioned in the advertisement to one of a general character. There are no allegations made by defendant affecting the moral character of the relator, and I feel constrained to consider his course at the time of his first establishing himself in Buffalo in the light of *youthful indiscretions* rather than unpardonable offences. The *locus penitentiœ* was not shut against him, and his abandonment of his emperical conduct for so long a time before his application, affords satisfactory evidence of an intent to avail himself of the right and duty to reform. While I have no doubt that the members of the Society who voted for his exclusion, were animated by a high sense of duty to their profession, yet they seem to me to have gone beyond their just powers in refusing him admission for acts which the relator had himself repudiated and abandoned.

If the relator should resume his obnoxious course after his admission he will be justly liable to the censure of the Society and ultimately to removal from its membership. But with the evidence of his present good conduct and of his adhesion to and intention to conform to their rules and ethics, he was legally entitled to admission to the Society, notwithstanding the professional errors of his early practice. The relator is entitled to the writ prayed for."

The President remarked that he should consider it his duty to notify Dr. Bartlett to sign the Constitution and By-Laws, etc., unless otherwise instructed by the Society.

After some discussion it was

*Voted* that the President be invited to communicate with the President of the State Medical Society, and after consultation with him, empowered to make appeal to the Term of the Supreme Court to be held in November, 1863, if he should think best.

*Voted* to adjonrn. J. F. Miner,

*Sec'y pro tem.*

